# Racial differences in the systemic inflammatory response to prostate cancer

**DOI:** 10.1371/journal.pone.0252951

**Published:** 2021-07-09

**Authors:** Andrew G. Rundle, Sudha M. Sadasivan, Dhananjay A. Chitale, Nilesh S. Gupta, Sean R. Williamson, Oleksandr N. Kryvenko, Yalei Chen, Kevin Bobbitt, Deliang Tang, Benjamin A. Rybicki

**Affiliations:** 1 Department of Epidemiology, Mailman School of Public Health, Columbia University, New York, NY, United States of America; 2 Department of Public Health Sciences, Henry Ford Hospital, Detroit, MI, United States of America; 3 Department of Pathology and Laboratory Medicine, Henry Ford Hospital, Detroit, MI, United States of America; 4 Department of Pathology and Laboratory Medicine, Department of Urology, and Sylvester Comprehensive Cancer Center, University of Miami Miller School of Medicine, Miami, FL, United States of America; 5 Department of Environmental Health Sciences, Mailman School of Public Health, Columbia Univ., New York, NY, United States of America; Brigham and Women’s and Harvard Medical School, UNITED STATES

## Abstract

Systemic inflammation may increase risk for prostate cancer progression, but the role it plays in prostate cancer susceptibility is unknown. From a cohort of over 10,000 men who had either a prostate biopsy or transurethral resection that yielded a benign finding, we analyzed 517 incident prostate cancer cases identified during follow-up and 373 controls with one or more white blood cell tests during a follow-up period between one and 18 years. Multilevel, multivariable longitudinal models were fit to two measures of systemic inflammation, neutrophil-to-lymphocyte ratio (NLR) and monocyte-to-lymphocyte ratio (MLR), to determine NLR and MLR trajectories associated with increased risk for prostate cancer. For both measures, we found no significant differences in the trajectories by case/control status, however in modeling NLR trajectories there was a significant interaction between race (white or Black and case-control status. In race specific models, NLR and MLR values were consistently higher over time among white controls than white cases while case-control differences in NLR and MLR trajectories were not apparent among Black men. When cases were classified as aggressive as compared to non-aggressive, the case-control differences in NLR and MLR values over time among white men were most apparent for non-aggressive cases. For NLR among white men, significant case-control differences were observed for the entire duration of observation for men who had inflammation in their initial prostate specimen. It is possible that, among white men, monitoring of NLR and MLR trajectories after an initial negative biopsy may be useful in monitoring prostate cancer risk.

## Introduction

The role of inflammation in prostate cancer is complex, and likely works at both a micro and macro level to influence prostate cancer initiation and progression [1]. At the cellular level, we and others have shown that histologically observable chronic and acute inflammation of the benign prostate may reduce the risk of subsequent prostate cancer [2–4]. However, a recent report from the Prostate Cancer Prevention Trial cohort study, of men without any indication for biopsy, suggests that histologic inflammation of the prostate may increase risk of disease [5]. Other evidence exists that suggests inflammation present at the time of detectable tumor may promote subsequent tumor growth leading to poor outcomes [6]. At the clinical level, manifestation of prostatitis appears to increase prostate cancer risk [7], although the consistency of this association is questionable particularly across different racial groups [8, 9]. The influence of inflammation on carcinogenesis goes beyond local inflammation at the site of tumorigenesis. Systemic inflammation can create a milieu conducive to cancer growth and can also be indicative of the body’s immune defense system reaction to early tumor growth [10].

The prostate is an immune-competent organ normally populated by inflammatory cells [11]. Neutrophils are one of the first responders of inflammatory cells during the beginning (acute) phase of inflammation, which can be caused by bacterial infection or an environmental insult. Cancer may be initiated at this phase, which can progress as inflammation persists and becomes chronic, where the primary immune cells become macrophages and lymphocytes [1]. Since acute and chronic inflammation can coexist, and there is no clear transition phase from one to the other, a full characterization of the inflammatory-response to cancer should include quantification of different inflammatory cell types. For example, the neutrophil- to-lymphocyte ratio (NLR) has become a clinically useful tool for predicting the response to therapy and prognosis in various types of malignancies, and may be a marker of a cancer-related environment [12, 13]. An elevated NLR indicates a high level of neutrophil-dependent inflammation with a concurrent reduction in the lymphocyte-mediated immune response, reflecting a carcinogenic milieu [14]. Studies of most cancers have also shown that tumor-infiltrating lymphocytes (TILs), likely recruited to eradicate cancer in its earliest stages, are associated with a good prognosis [15]. Recently in prostate cancer it was shown that a specific TIL phenotype based on cell surface markers predicted better survival outcomes after salvage radiotherapy [16]. Other white blood cell-based markers of inflammation, such as monocyte-to-lymphocyte ratio (MLR), have been less studied, and measure different features of the systemic inflammatory response. An increased MLR indicates a greater number of monocytes in circulation, which could indicate increased numbers of tumor associated macrophages and cancer progression [17]. Several studies have demonstrated that an elevated MLR is associated with poor cancer prognosis [18–21], including an association with high Gleason grade in prostate cancer [22].

Associations between inflammation and prostate may also vary by race. African American and Black men are at greater risk for prostate cancer [23], and gene expression profiles of prostate tumors indicate prominent differences in tumor immunobiology between African-American and European-American men [24]. One study has shown that histologic inflammation and higher PSA levels are more common in Black men with prostate cancer [25]. Another study of Black men found that prostatitis increased the risk for prostate cancer almost 5-fold [26], but a more recent study found no association in the Black case-control subset [8]. Our prior study of inflammation and prostate cancer risk found no racial differences in prevalence of inflammation in benign prostate [2], but we subsequently found clinical prostatitis was associated with a lower prostate cancer risk only among Black men [9].

In prostate cancer, measures of systemic inflammation have mainly been studied in the context of prostate cancer prognosis [27–29] and response to therapy [30]. More recently, MLR levels prior to diagnosis have shown to be associated with a prostate biopsy positive for cancer in men with modest PSA levels [31] and NLR was associated with Gleason upgrading [32]. No studies exist that have characterized changes in systemic inflammation before prostate cancer onset that may be indicative of early carcinogenesis. Studies of systemic inflammation and prostate cancer risk typically include a single pre-disease measure [33] and therefore no inference can be made as to whether the cancer-associated inflammatory response increases or decreases leading up to the time when a clinical diagnosis is made. Taking advantage of a unique cohort of men in a large health system identified through their benign prostate specimen [2], we used retrospective clinical laboratory data to determine whether longitudinal changes in systemic inflammation vary by race and if they could be used to discriminate between men who eventually are diagnosed with prostate cancer and those who remain cancer free in this high risk cohort.

## Methods

### Study sample

After obtaining approval from the Henry Ford Health System Institutional Review Board, we identified a cohort of 10,478 men with a benign prostate specimen collected between January 1990 and December 2012 with follow-up up to 18 years after cohort entry [2]. Using incidence density sampling within this cohort we then created a nested case-control study with 822 prostate cancer case-control pairs, with controls matched to cases on age at entry into cohort (±2 years), date of entry into cohort (±2 years), race (African American or White), and type of specimen (biopsy or transurethral resection of the prostate). Eligible cases were diagnosed a minimum of one year after cohort entry which provided an adequate window for repeat biopsies of suspicious findings (usually within six months of the initial biopsy) that resulted in a cancer diagnosis likely missed at initial biopsy. A prostate cancer diagnosis was confirmed by positive pathology or a clinical presentation consistent with prostate cancer (e.g. continued rising PSA that had reached 30 ng/ml or higher and/or evidence of bone metastasis). Eligible controls had one or more visits to the health system at a date that would put their observation period at least as long as the case to which they were matched.

### Statistical analysis

For descriptive analyses of case and controls presented in Table 1, p-values were calculated using chi square tests for dichotomous or categorical variables, using t-tests for continuous variables with approximately normal distributions and using non-parametric tests for non-normally distributed continuous variables.

**Table 1:** Case-control analytic dataset.

Variable	Cases: n = 517 Mean (SD) or %	Controls: n = 373 Mean (SD) or %	P value
Mean Age (SD)	64.9 (7.7)	65.4 (7.9)	0.3
Percent African American Race	46.6	42.4	0.2
Percent Biopsy^1^ Specimen	95.4	92.8	0.1
Mean years since cohort entry	6.3 (4.0)	7.2 (4.2)	0.002
Mean number of PSA tests	7.6 (5.6)	6.8 (6.2)	0.05
Mean number of WBC tests	3.6 (4.3)	4.2 (4.2)	0.08
Mean years between first and last WBC test^2^	4.3 (3.5)	4.7 (3.8)	0.06
Geometric mean monocyte-lymphocyte ratio^3^	0.31 (1.83)	0.30 (1.75)	0.32
Geometric mean neutrophil-lymphocyte ratio^3^	2.60 (1.97)	2.63 (1.96)	0.77
Percent Histologic prostatic inflammation	57.7	65.6	0.02
Mean PSA at cohort entry (ng/ml)	6.9 (5.9)	5.3 (4.6)	<0.0001

1 –remainder of specimens are transurethral resections of the prostate

2 –for subjects with 2 or more tests (N cases = 346; N controls = 266).

3 –Geometric mean and corresponding standard deviation based on results from earliest test after cohort entry

Data on all white blood cell tests undergone by study subjects during their follow-up period were retrieved from health system laboratory data downloaded from the electronic medical record. To focus on blood tests likely conducted as part of a routine exam or preventive health visit, the white blood cell data were filtered to remove sequences of temporally clustered blood tests that suggested an acute illness, hospitalization and/or that the study participant was under surveillance for an infection on an outpatient basis. To screen the data for clusters of blood tests, we identified all blood tests that occurred within 30 days of another blood test, which typically identified sequences of daily blood tests occurring over several days. For all blood tests occurring within 30 days of another blood test, we retained in the data set the last test identified in the 30-day window. In retaining the last test result we assumed that this test was indicative of the white blood cell measure at the end of an acute illness or period of surveillance. Two white blood cell measures were created: the ratio of neutrophils to lymphocytes (NLR) and the ratio of monocytes to lymphocytes (MLR). As these ratios were right skewed the ratio data were natural log transformed for analyses.

The men included in this analysis were originally selected to create a nested case-control study with controls individually matched to cases on duration of follow-up and confounders [34]. Nested case-control designs are used to identify exposures and/or participant characteristics measured at cohort enrollment that predict subsequent case-control status, but for the analyses presented here, case-control status was used to predict white blood cell ratios measured during the time between cohort entry and the case-control reference date [34, 35]. For cases, the date of diagnosis is the reference date, whereas for a matched control, the reference date was calculated by adding the follow-up time of the matched case to the cohort entry date of the matched control. The blood test dates were transformed so that the case-control reference dates were set to zero and the date of the blood tests was set to minus numbers indicating the number of days before the case-control reference date [36]. The data analysis strategy used mixed linear models to predict white blood cell ratios based on case-control status, time prior to the case-control reference date and interactions between time and case-control status, time squared and case-control status and time cubed and case-control status [35, 36]. The models were fit with random intercepts and random slopes and with blood tests clustered within men [37, 38]. Men with at least one eligible WBC measurement were included in the analyses: within this modeling framework, even a single value contributed information to estimating the mean MLR and NLR value at the time of blood test before the reference date. In instances when models failed to converge, the model specification of a random slope was removed. This modeling strategy allowed us to estimate and plot trajectories of white blood cell ratios in the years leading up to the case-control reference date [35]. Because of the zeroing of time at case-control reference date, the beta coefficient for case-control status estimates the difference in the natural log of the white blood cell ratios at the case-control reference date.

The initial mixed linear models specified clustering of cases and controls into their matched pair sets, however the analyses showed that clustering on the matched pairs did not account for any variance in the white blood cell ratios and this clustering was dropped from model specifications. Because the nested case-control design allows for men to be selected as both a control matched to an early case and then later in follow-up as a case, the initial data set included some subjects multiple times; men entered the data set when they were selected as controls and again later when they were selected as cases [34]. For several reasons, including: simplifying the data set; avoiding having the results of the same blood test represented in the data more than once; and because the initial analyses showed that incorporating the matching into the analyses provided no additional information, the data set was simplified so that each man was coded as a case or a control and appeared in the data set only once. If a control was matched to multiple cases (and therefore had multiple reference dates), his latest reference date was used in the analysis. This transformation of the data set removed the matching of the original design, and so key matching variables, age at diagnosis/control ascertainment and race, were included as covariates in the mixed linear model [35, 39]. In addition, the models controlled for PSA values at cohort entry, the presence or absence of inflammation in the initial benign prostate specimen and the presence of a clinical history of prostatitis. Because African Americans have lower NLR than Whites, out initial model included interaction terms between case-control status and race and case-control status and race and time. The interaction term between case-control status and race was significant and subsequent analyses were stratified by race. Covariate adjusted predicted mean values of NLR and MLR for cases and controls, and associated p-values for difference between cases and controls, were calculated from the models at the case/control reference date and each preceding six-month time point up to 5 years before the reference date. These predicted mean values were graphed to display trajectories of NLR and MLR for the five-year period prior to case diagnosis or control selection.

## Results

### Descriptive analyses

The initial white blood cell dataset consisted of 822 cases and 573 unique controls and 5,164 blood tests. Controls had no history of prostate cancer from the time of cohort entry to the last date of observation, which for controls matched to more than one case was their latest matched reference date. After filtering the white blood cell data as described in the methods section, our analytic data set comprised 3,427 unique WBC test results that included 1,879 test results on 517 cases and 1,548 test results on 373 controls. The number of WBC tests was highest in the first few years before diagnosis, given the study eligibility requirement of a minimum of one year of observation and most of the sample having three or more years of follow-up. In terms of frequency of WBC tests, cases had non-significantly fewer tests than controls; 3.64 compared to 4.15 (p = 0.08). Cases had a significantly higher PSA at cohort entry, significantly lower prevalence of prostatic inflammation, and a non-significantly higher number of PSA tests during follow-up (Table 1). Comparing the analytic sample to cases and controls without recorded WBC test results for analyses, we found prostate cancer cases and African Americans to be slightly overrepresented in the analytic sample (S1 Table) The follow-up time for men in the analytic sample was longer with a concomitant significantly higher number of PSA tests. Other significant differences between the analytic sample and those excluded from analysis include a lower percent of men with biopsy specimens, lower PSA level at cohort entry, and a higher percentage of stage 1 cancers among cases (S1 Table).

### Trajectory models

**Fig 1:**
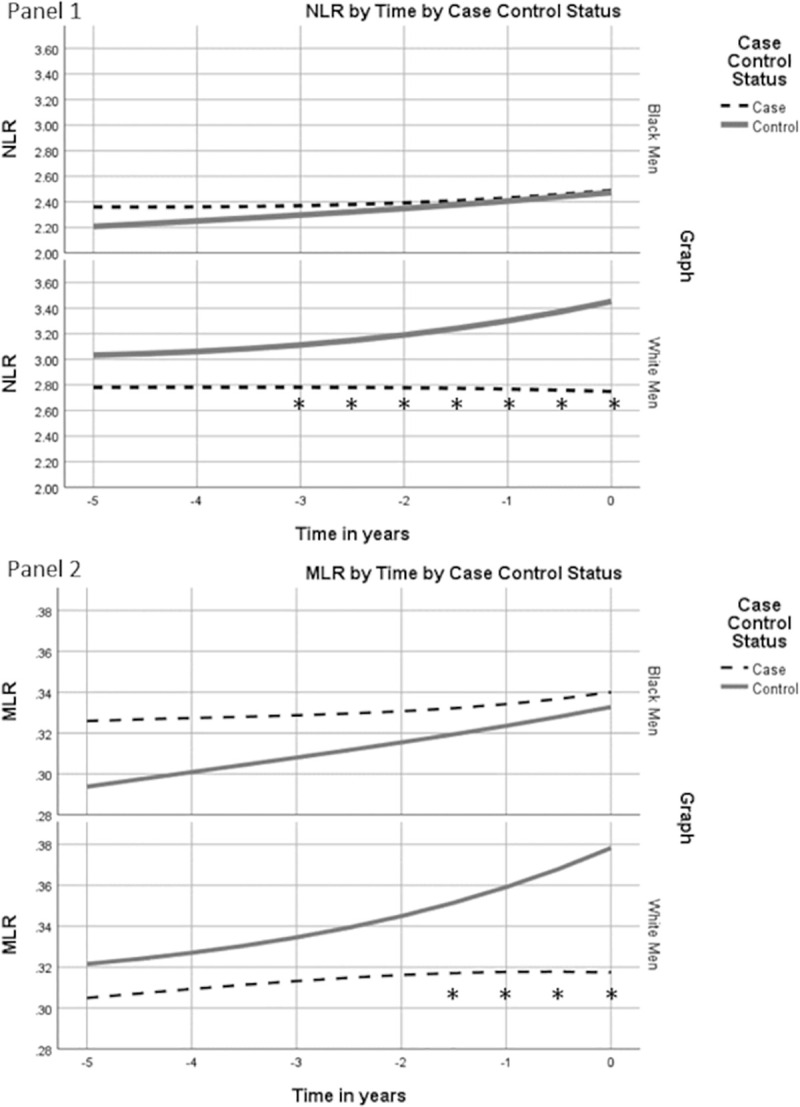
White blood cell ratios by case control status over time. Covariate adjusted geometric mean NLR and MLR plotted for cases and controls in years leading up to prostate cancer diagnosis or control ascertainment. * p<0.05 for case values compared to controls.

Race-stratified trajectory models for neutrophil-lymphocyte ratio (NLR) levels by case-control status are shown in Fig 1, panel 1. In white men, cases had a consistently lower NLR compared with controls during the five years leading up to diagnosis. In black men, while NLR levels were slightly and non-significantly higher among cases five years out from diagnosis, the NLR trajectories for cases and controls converged over time. For MLR trajectories (Fig 1, panel 2), differences between cases and controls among Black and White men were similar to the NLR trajectories: among white men MLR was consistently lower for cases compared to controls and among Black men MLR was initially higher among cases compared to controls, but the MLR trajectories converged over time.

**Fig 2:**
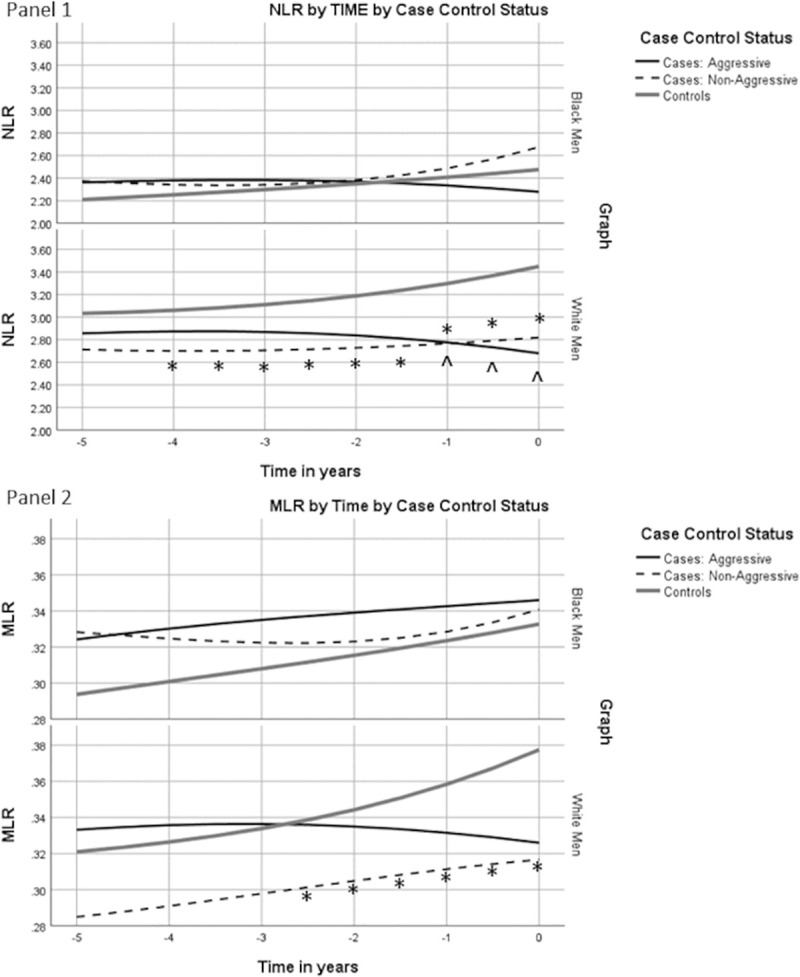
White blood cell ratios for cases with aggressive and non-aggressive disease and controls over time. Covariate adjusted geometric mean NLR and MLR plotted for cases with aggressive and non-aggressive disease and controls in years leading up to prostate cancer diagnosis or control ascertainment. ^ p<0.05 for values among case with aggressive disease compared to controls, * p<0.05 for values among cases with non-aggressive disease compared to controls.

We next examined NLR and MLR trajectories by case aggressiveness status (panel 1 and 2 of Fig 2). For NLR trajectories (Panel 1), stratifying by case aggressiveness did not alter the interpretation of case-control differences in NLR over time. Among white men, controls had a higher NLR over time than either men with aggressive disease at diagnosis or men with non-aggressive disease at diagnosis. For MLR, stratification of cases by disease aggressiveness at diagnosis revealed two different trajectories among white cases. White cases with non-aggressive disease consistently had lower MLR than white controls with an upward trajectory of MLR values over time in both groups. However, white cases with aggressive disease had MLR levels that were slightly higher than in controls 5 years prior to the reference date, however MLR levels among white aggressive cases slightly declined over time and was the same as non-aggressive cases at the time of diagnosis. Among Blacks, the trajectories of MLR were similar for cases with and without aggressive disease.

**Fig 3:**
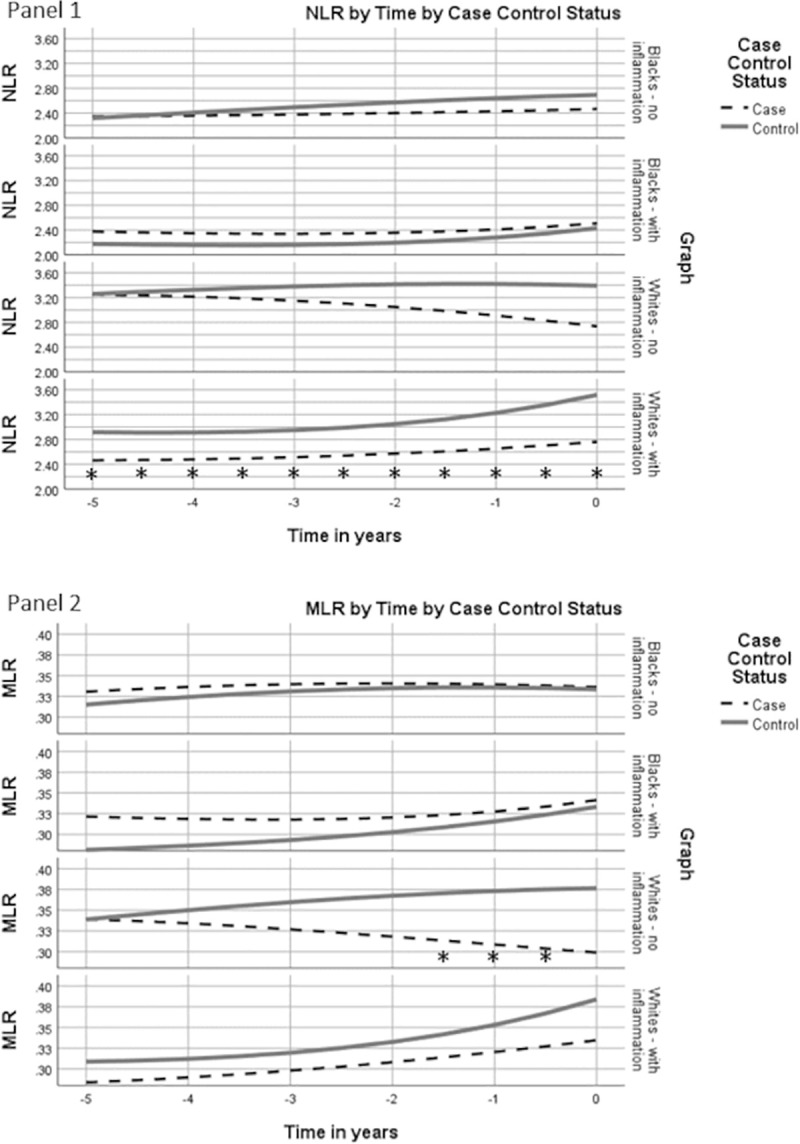
White blood cell ratios by case control status by prostatic inflammation status over time. Covariate adjusted geometric mean NLR and MLR plotted for cases and controls in years leading up to prostate cancer diagnosis or control ascertainment. * p<0.05 for case values compared to controls.

Prostatic inflammation is typically associated with benign prostatic hyperplasia, which is also related to markers of systemic inflammation, such as an increased WBC count [40]. Therefore, we reran NLR and MLR trajectory models stratifying men on the presence or absence of prostatic inflammation (Fig 3, panel 1 and 2). Among whites without inflammation, NLR and MLR values were equivalent in case and controls five years before the reference date, but NLR and MLR values among cases declined over time while values in controls increased slightly over time. About one year before diagnosis, MLR levels in cases were significantly less than in controls. For whites with prostatic inflammation, controls consistently had higher NLR and MLR values over time, with the differences for NLR being statistically significant for the entire time period. Among Blacks, NLR trajectories did not differ between case and controls regardless of inflammation status. For MLR values among Blacks, among those with inflammation, cases had non-significantly higher MLR than controls five years before diagnosis but MLR values among controls rose over time and were equivalent to cases by the time of diagnosis. Among blacks without inflammation, MLR values were essentially equivalent over time.

**Fig 4:**
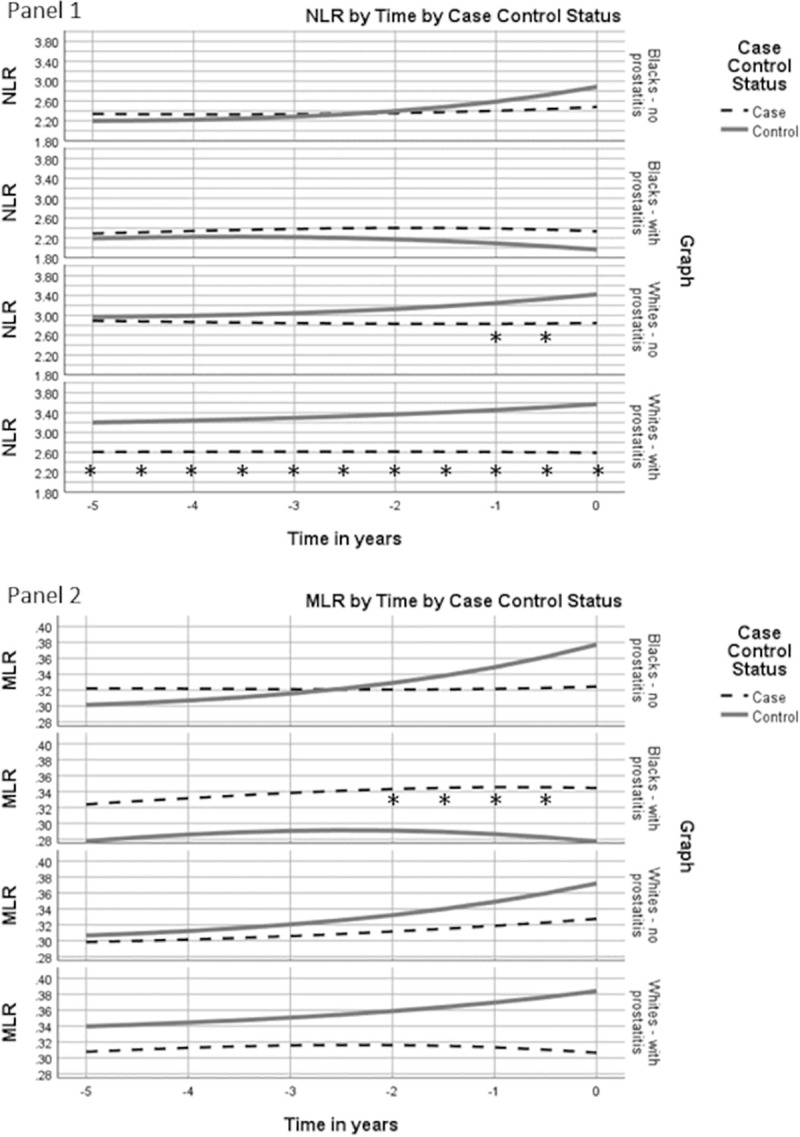
White blood cell ratios by case control status by history of prostatitis over time. Covariate adjusted geometric mean NLR and MLR plotted for cases and controls in years leading up to prostate cancer diagnosis or control ascertainment. * p<0.05 for case values compared to controls.

The data were also analyzed stratifying by the presence or absence of clinical prostatitis (Fig 4, panel 1 and 2). Among Blacks NLR values did not differ by case-control status regardless of prostatitis status. Among Whites with prostatitis, NLR values were significantly lower among cases than controls, while among Whites without prostatitis, NLR values were only significantly lower in the year before diagnosis. MLR values were higher for cases compared to controls among Blacks with prostatitis, with differences being significant between 6 months and 2 years before diagnosis. Among Whites with prostatitis, MLR values were non-significantly lower among cases compared to controls.

## Discussion

Systemic inflammation is thought to promote carcinogenesis and markers of systemic inflammation may be indicative of increased prostate cancer risk [41, 42]. However, in our study, among white men NLR and MLR values were consistently lower among cases compared to controls. For NLR this difference was more pronounced among men with a history of prostatitis, was exclusively observed among men with histologic evidence of prostatic inflammation and was more prominent for men with non-aggressive disease. For MLR, the overall difference for white men by case-control status was only significant in the 18 months before diagnosis, but when cancer was categorized as aggressive or non-aggressive, the difference in MLR values was confined to comparisons of non-aggressive cases and controls but was apparent beginning 2.5 years before diagnosis. Among White men with no evidence of histologic prostatic inflammation, MLR values among cases and controls diverged over time and values were significantly lower among cases in the 18 to 6 months before diagnosis. Among Black men no case-control differences were observed for NLR, and for MLR there were a few case-control differences, most notably among men with a history of prostatitis, cases had higher values than controls in the 18 to 6 months before diagnosis. The more robust differences we observed in the inflammatory response to prostate cancer development in white men compared with black men may be a key factor in determining race-specific targeted prevention strategies.

The interplay of race and inflammation in relation to prostate cancer risk is complex, and needs to be considered at several different levels. On a clinical level, prostatitis, as defined by symptomology related to prostate inflammation, appears to increase the risk for prostate cancer [7, 43, 44] although it may not be as strong a risk factor for prostate cancer in Blacks as compared to Whites [8, 9, 43]. However, a recent review strongly suggests that the association between clinical prostatitis and prostate cancer is largely driven by detection bias [45]. On a histological level, we previously found prostatic inflammation in both White and Black men was associated with a decreased risk for prostate cancer [2]. While others have reported similar reduced prostate cancer risk associated with histologic inflammation of the prostate [3, 46, 47], to our knowledge no other studies have specifically examined the cancer risk of Black men with prostatic inflammation. Eastham et al. showed that among men under suspicion of prostate cancer, but found biopsy negative, a higher prevalence of histologic inflammation exists in Black men, but they did not specifically examine the association between histologic inflammation and prostate cancer risk [25]. On a molecular level, several groups have shown a distinct immune signature in prostate tumors of Black men [24, 48]. In terms of systemic inflammation, both the underlying genetics of inflammation [49, 50] and markers of systemic inflammation appear to differ by race [51, 52]. Among Black men, lower NLR levels have been consistently reported [53, 54], and were also apparent in this study.

A robust literature exists for the use of both NLR and MLR as markers of disease prognosis in prostate cancer. Overall, it appears that when these markers are elevated there is a greater risk for poor disease outcome [28, 29, 55, 56]. In terms of NLR, which is the most tested inflammatory marker of disease prognosis, this biomarker seems to be more predictive of advanced prostate cancer compared with local disease. Interestingly, among white men in our study population, the trajectories of NLR and MLR showed consistently lower values among non-aggressive cases compared to controls. While for aggressive cases both NLR and MLR trajectories deflected downwards in the two years prior to diagnosis, with NLR values becoming significantly lower than values in controls in the year before diagnosis. This is in contrast to reports that have shown both increased NLR [57] and monocyte counts [58] are associated with higher grade disease.

Only a few studies have examined NLR and MLR as a marker of prostate cancer risk and none have examined trajectories of these markers leading up to diagnosis. Both elevated [59] and decreased [60] NLR levels have been associated with greater risk of prostate cancer at time of biopsy with some reports finding no association [61, 62]. Other studies have found elevated NLR to be predictive of prostate cancer only in subsets of patients based on low or high PSA levels [63, 64]. Our study finds that for white men NLR and MLR values are lower among cases than controls. Only one other study has examined racial differences in the predictive ability of inflammatory markers in prostate cancer. Vidal et al. found that NLR was not predictive of disease outcomes in either white or black men undergoing prostatectomy, but did find a neutrophils-positive association with risk of all-cause mortality in white men [65].

Analyses of systemic markers of inflammation as risk factors for prostate cancer should also consider prostatic inflammation. White blood cell count may be associated with the degree of prostate enlargement [40] and lower urinary tract symptoms and elevated NLR levels are associated with LUTS severity in patients with BPH [66], suggesting a direct correlation between this marker and prostatic inflammation. Most investigators that have examined NLR in relation to prostate cancer risk or progression have not taken into account prostatic inflammation. One exception was a study of South Korean men with initial prostate-specific antigen (PSA) levels ranging from 4 to 10 ng ml−1 who underwent TRUS-guided prostate biopsy–patients with a history of prostatitis were excluded and the investigators subsequently found NLR was significantly associated with prostate cancer detection [64]. All of the analyses presented here controlled for the presence of histologic prostatic inflammation, but when cases and controls were stratified by the presence or absence of inflammation, the overall lower NLR values seen in white cases compared to controls was most apparent for men whose initial benign prostate specimen showed inflammation. Among White cases with no prostatic inflammation, NLR trended downwards over the observation period but did not significantly differ from levels observed in controls.

In our large, racially diverse study sample with long follow-up, the availability of longitudinal data from clinical WBC tests allowed us to model the trajectories of systemic markers of inflammation. Clinical tests were not done systematically, but rather based on indication, which limits their utility for research purposes. However, we were careful to prune out excessive WBC tests indicative of disease processes that may have increased inflammation in an acute manner, and therefore the resulting tests should better reflect longitudinal changes in NLR and MLR levels and not overly influenced by acute disease events. However, we cannot be certain that all WBC tests related to an acute disease episode or surveillance of a suspected disease were pruned from the data set, and this is a weakness of the study. Since our study cohort was based on having a benign prostate specimen, it cannot be considered representative of all older men at risk for prostate cancer. However, given that inflammatory processes were probably overrepresented in our high-risk study cohort, any finding of any association between prostate cancer and increasing levels of an inflammatory marker, such as NLR, is unlikely to be a spurious finding. The clinical nature of the WBC tests also excluded a high percentage of the original case-control sample from the analytic dataset. In general, it appeared that the analytic sample was at a lower risk for prostate cancer compared with those excluded from analysis given the lower PSA level at cohort entry and longer observation periods in the analyzed subset. However, for the results presented here to be spurious, the NLR and MLR values would have to be systematically different by race, case-control status, tumor aggressiveness and initial biopsy specimen inflammation status among men included in the analytical sample compared to those men excluded.

In conclusion, NLR and MLR values were consistently higher among white controls than white cases while case-control differences were not particularly apparent among black men. When cases were classified as aggressive as compared to non-aggressive, the case-control differences in NLR and MLR values among white men was most apparent for non-aggressive cases. For NLR among white men, significant case-control differences were apparent for the entire duration of observation for men who had evidence of histologic prostatic inflammation. The finding that markers of systemic inflammation are lower among cases than controls among whites echoes our prior finding that benign prostatic inflammation is protective against future prostate cancer risk. It is possible that monitoring of NLR and MLR after an initial negative biopsy may be useful in monitoring prostate cancer risk.

## Supporting information

S1 Table Comparison of case-control analytic dataset with excluded cases and controls on selected demographic and clinical characteristics. (DOCX)
