# Supplementary material for: Racial differences in the systemic inflammatory response to prostate cancer
Source: PLoS One. 2021 Jul 9;16(7):e0252951. doi: 10.1371/journal.pone.0252951 (PMC8270440; doi:10.1371/journal.pone.0252951)
Supplement: S1 Table — (DOCX) [file pone.0252951.s001.docx]

S1 Table. Comparison of case-control analytic dataset with excluded cases and controls on selected demographic and clinical characteristics

| Variable | In Study: n=890  Mean (SD) or % | Excluded from Study: n=469  Mean (SD) or % | P-Value |
| --- | --- | --- | --- |
| Age | 65.1 (7.8) | 64.4 (7.8) | 0.1 |
| Percent African  American Race | 44.8 | 40.0 | 0.07 |
| Percent who were  Prostate Cancer Cases | 58.1 | 65.0 | 0.01 |
| Percent with a Biopsy  Specimen^1^ | 94.3 | 97.0 | 0.02 |
| Years since cohort entry | 6.7 (4.1) | 3.8 (2.6) | <0.0001 |
| Number of PSA tests | 7.3 (5.9) | 4.2 (3.9) | <0.0001 |
| Percent with Histologic  prostatic inflammation | 61.0 | 57.3 | 0.2 |
| PSA at cohort entry (ng/ml) | 6.2 (5.4) | 7.1 (7.4) | 0.02 |
| Mean PSA at Diagnosis^2^ | 24.7 (182.5) | 26.9 (198.2) | 0.9 |
| Tumor grade^2^ |  |  |  |
| 1 | 50.6 | 50.8 | 0.8 |
| 2 | 22.0 | 21.6 |  |
| 3 | 10.3 | 9.6 |  |
| 4 | 10.3 | 12.6 |  |
| 5 | 6.8 | 5.3 |  |
| Tumor Stage^2^ |  |  |  |
| 1 | 46.4 | 36.7 | <0.0001 |
| 2 | 44.4 | 51.0 |  |
| 3 | 8.3 | 11.3 |  |
| 4 | 1.0 | 1.0 |  |

1 – Remainder of specimens were transurethral resections of the prostate

2 – Cases only (n=517 and 305)
